# TIPE1 suppresses invasion and migration through down‐regulating Wnt/β‐catenin pathway in gastric cancer

**DOI:** 10.1111/jcmm.13362

**Published:** 2017-10-10

**Authors:** Wenwen Liu, Ye Chen, Hua Xie, Yongmin Guo, Dandan Ren, Yupeng Li, Xu Jing, Dongliang Li, Xiao Wang, Miaoqing Zhao, Tianfeng Zhu, Ziying Wang, Xinbing Wei, Fei Gao, Xiaojie Wang, Suxia Liu, Yan Zhang, Fan Yi

**Affiliations:** ^1^ Department of Pharmacology Shandong University School of Medicine Jinan China; ^2^ Taishan District Center for Disease Control and Prevention Taian China; ^3^ Department of Anesthesiology Qilu Hospital of Shandong University Jinan China; ^4^ Department of Pediatrics Peoples Hospital of Rizhao Rizhao China; ^5^ Department of Pathology Shandong University School of Medicine Jinan China; ^6^ Department of Pathology Shandong Provincial Hospital Shandong University Jinan China; ^7^ Key Laboratory of Cardiovascular Remodeling and Function Research Chinese Ministry of Education and Chinese Ministry of Health Qilu Hospital Shandong University Jinan China; ^8^ Department of Immunology Shandong University School of Medicine Jinan China

**Keywords:** TIPE1, gastric cancer, epithelial–mesenchymal transition, Wnt/β‐catenin pathway

## Abstract

Epithelial–mesenchymal transition (EMT) plays an important role in the invasiveness and metastasis of gastric cancer. Therefore, identifying key molecules involved in EMT will provide new therapeutic strategy for treating patients with gastric cancer. TIPE1 is a newly identified member of the TIPE (TNFAIP8) family, and its contributions to progression and metastasis have not been evaluated. In this study, we found that the levels of TIPE1 were significantly reduced and inversely correlated with differentiation status and distant metastasis in primary gastric cancer tissues. We further observed overexpression of TIPE1 in aggressive gastric cancer cell lines decreased their metastatic properties both *in vitro* and *in vivo* as demonstrated by markedly inhibiting EMT and metastasis of gastric cancer cells in nude mice. Consistently, gene silencing of TIPE1 in well‐differentiated gastric cancer cell line (AGS) inhibited these processes. Mechanistically, we found that TIPE1‐medicated Wnt/β‐catenin signalling was one of the critical signal transduction pathways that link TIPE1 to EMT inhibition. Importantly, TIPE1 dramatically restrained the expression and activities of MMP2 and MMP9 which are demonstrated to promote tumour progression and are implicated in EMT. Collectively, these findings provide new evidence for a better understanding of the biological activities of TIPE1 in progression and metastasis of gastric cancer and suggest that TIPE1 may be an innovative diagnostic and therapeutic target of gastric cancer.

## Introduction

Gastric cancer is the fourth most common cancer and the second leading cause of cancer‐related death worldwide 1, 2. Despite studies have seen dramatic advances in the understanding of the pathogenesis of gastric cancer, there is no effective therapy available for the treatment of gastric cancer. Therefore, it is urgent to identify novel molecular markers and explore related molecular mechanisms contributing to improving diagnostic and therapeutic management of gastric cancer. The main reasons for failure and mortality of gastric cancer are cell infiltration and metastasis. Mounting evidence has indicated that EMT plays an important role in the invasiveness and metastasis of gastric cancer cells by the dissolution of cell–cell junctions and the development of individual motile mesenchymal cells with increased mobility 3, 4. In addition, during EMT, gastric cancer cells can also obtain the stem cell properties which are closely associated with tumour relapse 5. Therefore it is essential to unveil the underlying mechanism for EMT, which is instrumental in understanding the cause for tumour malignant progression.

TIPE1 (tumour necrosis factor‐α‐induced protein 8‐like 1 or TNFAIP8L1) is a newly identified member of the TIPE (TNFAIP8) family, which consists of four members: TNFAIP8, TIPE1, TIPE2 and TIPE3. Although all the members of TIPE family share similar sequence and structures, they have distinct biologic functions. TNFAIP8 and TIPE3 are of importance in oncogenesis 6, 7. TIPE2 serves as an essential negative regulator of immunity and plays important roles in inflammatory diseases 8, 9. However, the biologic functions of TIPE1 in the physiological and pathologic conditions are largely unknown. A very recent study indicates that TIPE1 induces apoptosis by negatively regulating Rac1 activation in hepatocellular carcinoma cells 10. However, whether TIPE1 is involved in gastric cancer progression and metastasis keeps unknown. This study was designed to investigate the potential role of TIPE1 in gastric cancer invasion, progression and metastasis. We found that TIPE1 was inversely correlated with differentiation status and distant metastasis in primary gastric cancer tissues and further demonstrated that TIPE1 functions as a metastasis suppressor in gastric cancer by suppressing Wnt/β‐catenin signalling and MMP activity, suggesting that TIPE1 may be a new prognostic indicator and therapeutic target of gastric cancer.

## Materials and methods

Cell transfection, RNA extraction and real‐time RT‐PCR, Western blot analysis, immunohistochemistry and cell migration and invasion assays were presented as online supplemental materials.

### Patients

The 102 clinical specimens were obtained from Qilu Hospital of Shandong University (Jinan, China) during year of 2013–2015, which were approved by the ethics committee of Shandong University (No.LL‐201201056). The work described has been carried out in accordance with The Code of Ethics of the World Medical Association. The 102 patients were diagnosed with primary gastric cancer and none of them received chemotherapy or radiotherapy before surgery. The samples were collected immediately after surgery. The diagnosis of gastric cancer for all patients was confirmed by pathological examination, and the pathologic tumour –node–metastasis (TNM) status was assessed according to the criteria of the seventh edition of American Joint Committee on Cancer/International Union against Cancer TNM classification system. The related pathologic and clinic features of patients are listed in Table 1.

**Table 1:** Correlation between TIPE1 expression and clinicopathologic features in gastric cancer specimens

Feature	Total	TIPE1 expression^a^		
		Low	High	χ^2^ value and *P*
Age				
<60	52	26 (50.0%)	26 (50.0%)	χ^2^ * *=* *0.163 *P *=* *0.686
≥60	50	23 (46.0%)	27 (54.0%)	
Sex				
Male	74	32 (43.2%)	42 (56.8%)	χ^2^ * *=* *2.484 *P *=* *0.115
Female	28	17 (60.7%)	11 (39.3%)	
Tumour differentiation				
High‐differentiated tubular adenocarcinoma	14	3 (21.4%)	11 (78.6%)	χ^2^ * *=* *15.164 *P *=* *0.001
Moderate‐differentiated tubular adenocarcinoma	29	8 (27.6%)	21 (72.4%)	
Poorly cohesive carcinoma	59	38 (64.4%)	21 (35.6%)	
pT Status				
1	17	9 (52.9%)	8 (47.1%)	χ^2^ * *=* *6.981 *P *=* *0.030
2	19	8 (42.1%)	11 (57.9%)	
3~4	66	15 (22.7%)	51 (77.3%)	
pN Status				
0	44	15 (34.1%)	29 (65.9%)	χ^2^ * *=* *8.339 *P *=* *0.040
1	29	17 (58.6%)	12 (41.4%)	
2	23	13 (56.5%)	10 (43.5%)	
3	6	5 (83.3%)	1 (16.7%)	
pM Status				
pM0	91	39 (42.9%)	52 (57.1%)	χ^2^ * *=* *9.078 *P *=* *0.003
pM1	11	10 (90.9%)	1 (9.1%)	
pTNM Stage				
I	22	7 (31.8%)	15 (68.2%)	χ^2^ * *=* *9.600 *P *=* *0.022
II	43	18 (41.9%)	25 (58.1%)	
III	25	14 (56.0%)	11 (44.0%)	
IV	12	10 (83.3%)	2 (16.7%)	

a Semi‐quantitative analyses of TIPE1 expression using real‐time PCR and relative abundances of each sample were normalized with normal tissues.

### Animal studies

Male nude mice (5–6 weeks old) were purchased from Beijing Vital River Laboratory Animal Technology Co., LTD (Beijing, China) and divided into four groups. 7 × 10^5^ BGC‐823 cells (or 10 × 10^5^ SGC‐7901 cells) were injected into nude mice through tail vein. One group was injected into gastric cancer cells with the overexpression of TIPE1, and the other group was injected into the matched control cells. One and a half month later, the mice were killed and the organs were harvested and photographed. The animal experimental protocols were approved by Institutional Animal Care and Use Committee of Shandong University and conducted in accordance with the National Institutes of Health (NIH) Guide for the Care and Use of Laboratory Animals (Permit number: LL‐201201056).

### Cell culture

Human gastric adenocarcinoma cell lines AGS, BGC‐823 and SGC‐7901 were obtained from Cancer Institute of Beijing (Beijing, PR China) free of mycoplasma. Cells were cultured at 37°C with 5% CO_2_ in RPMI 1640 (SGC7901, BGC‐823) or DMEM F‐12 (AGS) medium (Hyclone, Thermo, Beijing, China) containing 10% foetal bovine serum (FBS), 100 U/ml penicillin and 2 mmol/l L‐glutamine.

### Microarray analysis

The microarray experiments were performed by Shanghai Biotechnology Corporation, Shanghai, China. The submitted sample was fresh gastric cancer tissue (poorly cohesive carcinoma) and adjacent non‐tumour tissue from the same gastric cancer patient.

### Wound‐healing assay

Wound‐healing assay was performed as described 4.

### Gelatin zymography assay

Gelatin zymography assays were performed as described using commercial kit (MMP zymography electrophoretic analysis kit, GENMED, Shanghai) 11. Densitometry was used to quantify the MMP bands.

### Statistical analysis

Student's *t*‐test (two‐tailed), one‐way anova and the Χ^2^ test were used to analyse data, along with SPSS 16.0 (IBM, Chicago IL, USA). *P* values of <0.05 were considered statistically significant.

## Results

### The levels of TIPE1 were significantly reduced and inversely correlated with differentiation status and distant metastasis in primary gastric cancer tissues

**Figure 1:**
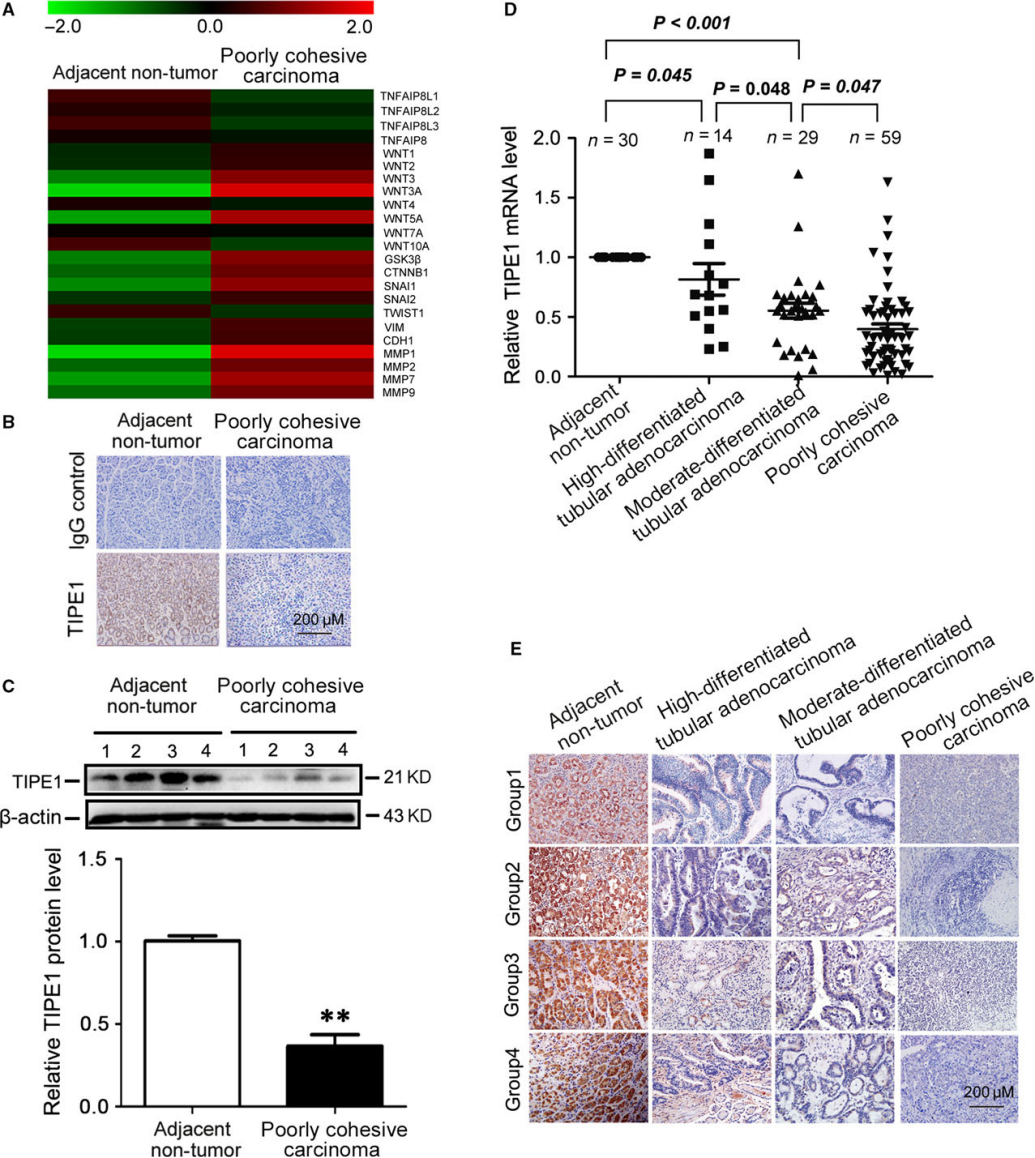
TIPE1 was significantly reduced in primary gastric cancer tissues (**A**) Representative heatmap of gene expression levels including TIPE1 family and related downstream genes by multiplex qRT‐PCR array. (**B**) Representative photomicrographs of TIPE1 immunohistochemical staining in the adjacent non‐tumour tissues and poorly cohesive carcinoma tissues. (**C**) Representative Western blot gel documents and summarized data showing the protein levels of TIPE1 in the adjacent non‐tumour tissues and poorly cohesive carcinoma tissues. (**D**) Relative mRNA levels of TIPE1 in gastric cancer tissues and the adjacent non‐tumour tissues. (**E**) Representative photomicrographs of TIPE1 immunohistochemical staining in the adjacent non‐tumour tissues and the gastric cancer tissues. **P <* *0.01, *versus* adjacent non‐tumour tissues (*n* = 6).

First, we examined the expression patterns of TIPE family in primary gastric cancer specimens by Agilent Whole Human Genome Oligo Microarray for global gene expression analysis (Fig. 1A). It was found that the level of TIPE1 and TIPE3 was decreased in poorly cohesive carcinoma compared with adjacent non‐tumour tissue. The microarray data have been submitted to GEO (ID number: GSE99908). The reduction of TIPE1 in poorly cohesive carcinoma was further confirmed by immunohistochemical staining (Fig. 1B) and Western blot analyses (Fig. 1C). We further analysed the expression of TIPE1 from 102 cases of primary gastric cancer specimen stratified by TNM stage, distant metastasis, tumour location and degree of gastric cancer cell differentiation. We found that the decreased levels of TIPE1 were associated with degree of gastric cancer cell differentiation by mRNA analysis (Fig. 1D) and immunohistochemical staining (Fig. 1E). χ^2^ test further demonstrated no distinguished relationship among TIPE1 expression, the patients' age and gender (*P *>* *0.05). However, the expression of TIPE1 was inversely correlated with differentiation status (*P *=* *0.001), aggregated degree (*P *=* *0.030), lymph node status (*P *=* *0.040), distant metastasis (*P *=* *0.003) and TNM stage (*P *=* *0.022) of patients with gastric cancer (Table 1).

### TIPE1 suppressed EMT in gastric cancer cells

**Figure 2:**
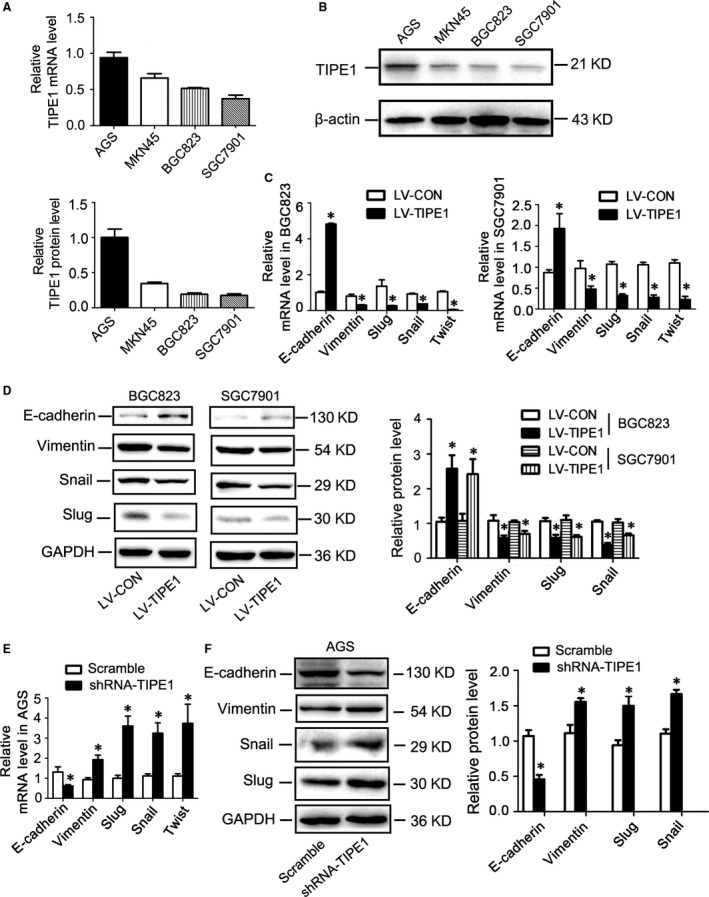
TIPE1 suppressed EMT in gastric cancer cells (**A**) Relative mRNA levels of TIPE1 in a panel of gastric cancer cells lines. (**B**) Representative Western blot gel documents and summarized data showing the levels of TIPE1 in a panel of gastric cancer cells lines. (**C**) Relative mRNA levels of epithelial and mesenchymal markers in BGC823 and SGC7901 cells. (**D**) Representative Western blot gel documents and summarized data showing the protein levels of epithelial and mesenchymal markers in BGC823 and SGC7901 cells. (**E**) Relative mRNA levels of epithelial and mesenchymal markers in AGS. (**F**) Representative Western blot gel documents and summarized data showing the protein levels of epithelial and mesenchymal markers in AGS cells. *P <* *0.05, *versus* LV‐CON groups (*n* = 6).

To study whether TIPE1 is associated with degree of tumour cell differentiation, we surveyed the expression levels of TIPE1 in a panel of gastric cancer cell lines that are derived from tumours with various degrees of differentiation. Our results showed that the levels of TIPE1 were lower in low‐differentiated gastric cancer cell lines including SGC7901, BGC823 and MKN45 than those of well‐differentiated AGS cell lines (Fig. 2A–B). EMT is a vital biological process involved in cell differentiation in normal embryonic development and is a potential mechanism for tumour cell metastasis. To address whether TIPE1 has effects on EMT, we overexpressed TIPE1 using lentivirus in BGC823 and SGC7901 cells (Fig. S1A–D) or knocked down TIPE1 expression in AGS (Fig. S1E). The expression of the epithelial marker such as E‐cadherin and mesenchymal markers was determined. Although overexpression of TIPE1 did not change the cell morphology (Fig. S2), the epithelial cell markers, E‐cadherin, were up‐regulated compared with the controls, whereas mesenchymal cell marker vimentin and transcription factors that are known to promote EMT such as Snail, Slug and Twist were significantly down‐regulated in BGC823 and SGC7901 cells by mRNA (Fig. 2C) and Western blot (Fig. 2D) analyses, which was further confirmed by immunofluorescence staining in BGC823 cells (Fig. S3). Furthermore, gene silencing of TIPE1 reversed EMT in AGS (Fig. 2E and F).

### TIPE1 inhibited migratory and invasive capacities in gastric cancer cells

**Figure 3:**
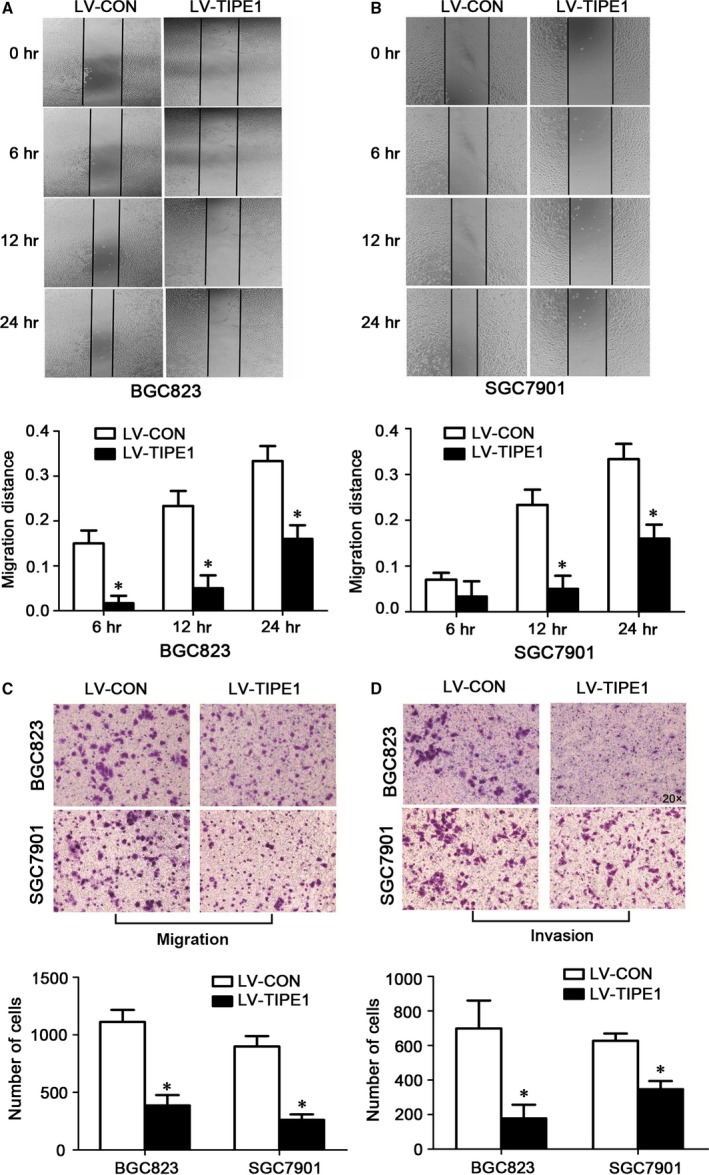
TIPE1 inhibited migratory and invasive capacities in gastric cancer cells (**A**) Representative and quantification of wound‐healing assay in BGC823 cells. (**B**) Representative and quantification of wound‐healing assay in SGC7901 cells. (**C**) Representative matrigel assay of the migration in BGC823 and SGC7901 cells and summarized data showing the number of cells passing through the Matrigel filter. (**D**) Representative matrigel assay of invasion in BGC823 and SGC7901 cells and summarized data showing the number of cells passing through the Matrigel filter. *P <* *0.05, *versus* LV‐CON groups (*n* = 6).

By wound‐healing assay, we found that overexpression of TIPE1 in BGC823 and SGC7901 cells had significantly slower closure of the wound area compared to their controls (Fig. 3A–B), which was further confirmed by transwell (Fig. 3C) and matrigel invasion assays (Fig. 3D) indicating that TIPE1 regulated migratory and invasive behaviours of gastric cancer cells. Consistently, gene silencing of TIPE1 in AGS cells showed the relative low migratory and invasive capacities (Fig. S4A–B).

### TIPE1 ameliorated tumourigenesis and colonization of gastric cancer cells

**Figure 4:**
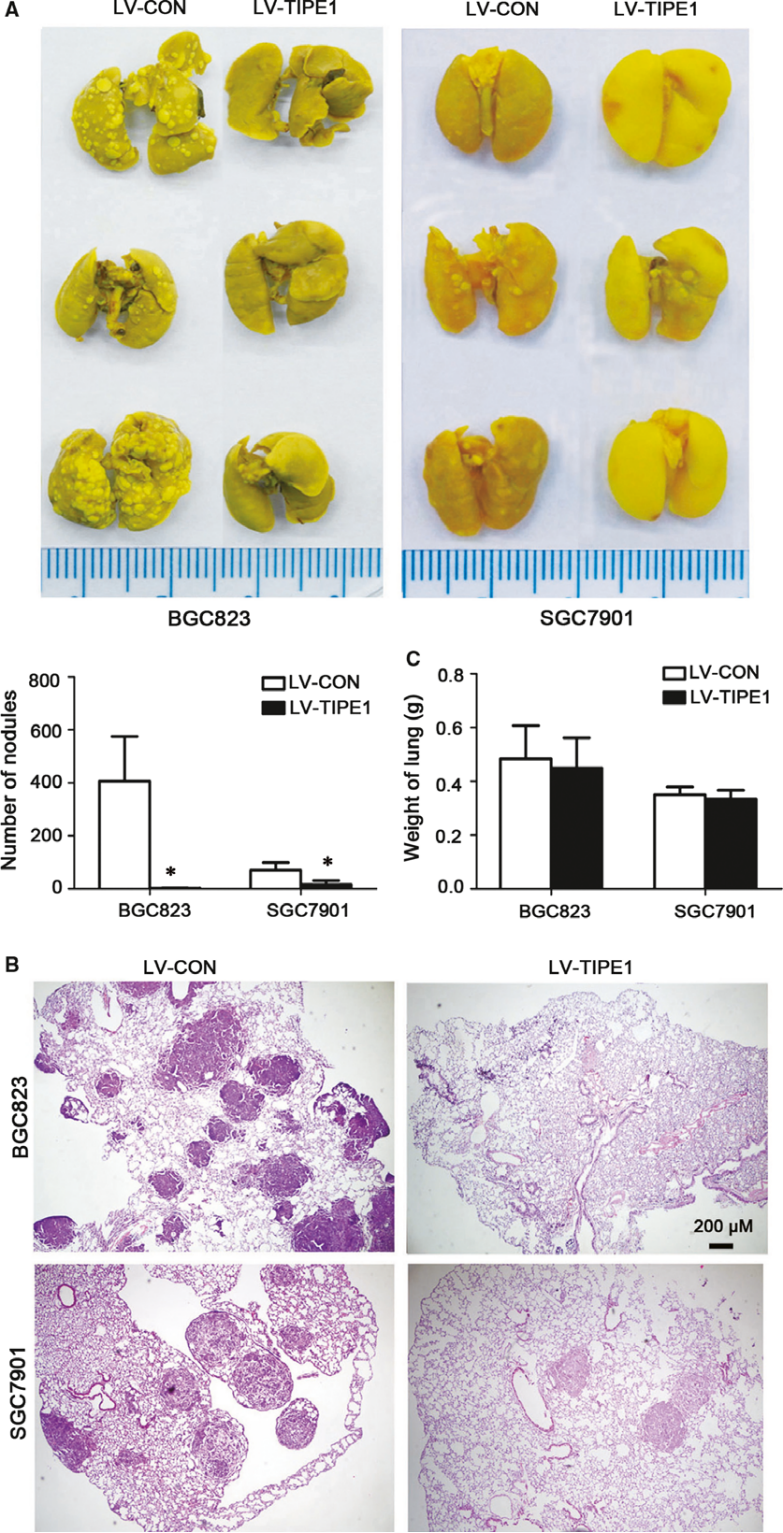
TIPE1 ameliorated tumourigenesis and colonization of gastric cancer cells (**A**) Representative metastatic nodules in lungs by picric acid staining and summarized data showing the number of metastatic nodules. (**B**) Representative haematoxylin and eosin staining of lung tissues. (**C**) Summarized data showing the weight of lungs. *P <* *0.05, *versus* LV‐CON groups (*n* = 10).

We injected BGC823 cells and SGC7901 cells infected with lentivirus‐TIPE1 into the tail vein of nude mice and examined their internal organs for tumours seeding. We found that the number of metastatic nodules in lungs was significantly reduced by lentivirus‐TIPE1 as shown in Figure 4A–B by picric acid and HE staining, although no statistical significance in lung weight was observed among these groups (Fig. 4C).

### TIPE1 restrained the expression and activities of MMP2 and MMP9

**Figure 5:**
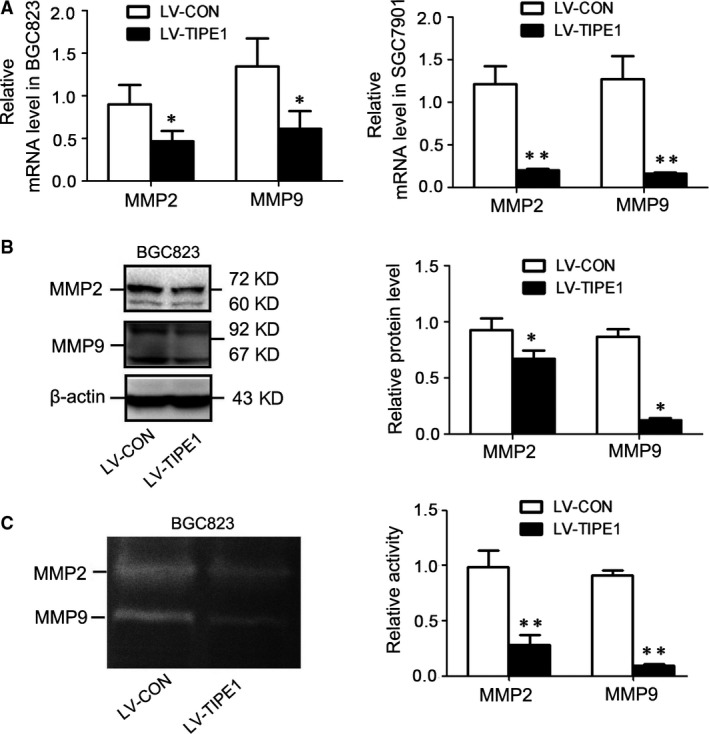
TIPE1 restrained the expression and activities of MMP2 and MMP9 (**A**) Relative mRNA levels of MMP2 and MMP9 in BGC823 and SGC7901 cells. (**B**) Representative Western blot gel documents and summarized data showing the expression levels of MMP2 and MMP9 in BGC823 cells. (**C**) Representative gelatin zymography assay in BGC823 cells and summarized data showing the activities of MMP2 and MMP9 in BGC823 cells. *P <* *0.05, **P <* *0.01, *versus* LV‐CON groups (*n* = 6).

Previous studies indicated that the elevated expression of MMP2 and MMP9 correlated with the invasive capability of gastric cancer cells. Therefore, we detected the role of TIPE1 on the expressions of MMP2 and MMP9 and found that TIPE1 reduced the expressions of MMP2 and MMP9 in BGC823 and SGC7901 cells by mRNA (Fig. 5A) and Western blot (Fig. 5B) analyses. Gelatin zymography assay further indicated the activity of MMP2 and MMP9 was restrained by TIPE1 in BGC823 cells (Fig. 5C), which may partially explain that TIPE1 can inhibit migratory and invasive behaviours in gastric cancer cells.

### TIPE1 negatively regulated the Wnt/β‐catenin signalling pathway

**Figure 6:**
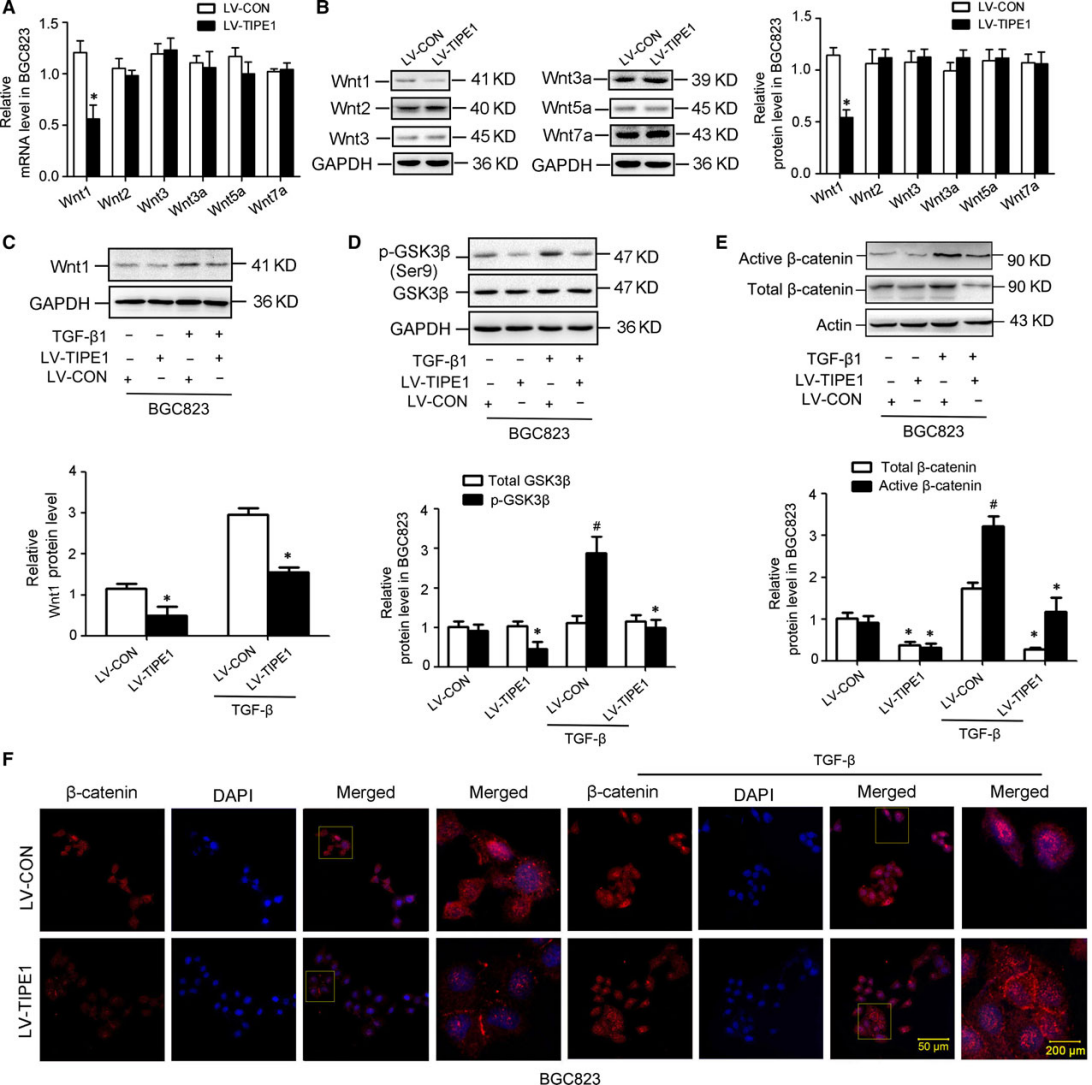
TIPE1 negatively regulated Wnt/β‐catenin signalling pathways. (**A**) Relative mRNA levels of Wnt1, Wnt2, Wnt3, Wnt3a, Wnt5a and Wnt7a in BGC823 cells. (**B**) Representative Western blot gel documents and summarized data showing the expression levels of Wnt1, Wnt2, Wnt3, Wnt3a, Wnt5a and Wnt7a in BGC823 cells. (**C**) Representative Western blot gel documents and summarized data showing the expression levels of Wnt1 in BGC823 cells with different treatments. (**D**) Representative Western blot gel documents and summarized data showing the expression levels of total and phosphorylated GSK3β protein in BGC823 cells. (**E**) Representative Western blot gel documents and summarized data showing the expression levels of active and total β‐catenin protein in BGC823 cells. (**F**) Immunofluorescence staining results showing the distribution of β‐catenin in the BGC823 cells. *P <* *0.05, ^#^ *P *<* *0.05 *versus* LV‐CON groups (*n* = 6).

Wnt/β‐catenin pathway is one of the major signalling pathways involved in EMT and plays a critical role in metastasis. Our results showed that TIPE1‐negative regulated Wnt1 expression, but had no effects on the expression of other major Wnt isoforms such as Wnt2, Wnt3, Wnt3a, Wnt5a and Wnt7a by mRNA (Fig. 6A) and Western blot (Fig. 6B) analyses. To examine the biologic consequence of Wnt induction, we next investigated the activation of GSK3β and β‐catenin, the canonical pathway of Wnt signalling in BGC823 cells in response to TGF‐β1. We found that overexpression of TIPE1 attenuated TGF‐β1‐induced Wnt1 expression (Fig. 6C). Compared with control, the amount of phosphorylated GSK3β (Fig. 6D) and active β‐catenin (dephosphorylated β‐catenin,Fig. 6E) was dramatically reduced by TIPE1 as well as the total β‐catenin expression levels. Consistently, immunofluorescence staining further confirmed that TIPE1 significantly decreased the expression of β‐catenin and induced more β‐catenin distributing to the membrane in BGC823 cells (Fig. 6F). In addition, gene silencing of TIPE1 in AGS cells increased Wnt1‐mediated the activity of Wnt/β‐catenin signalling (Fig. S4C–F). However, inhibition of Wnt/β‐catenin signalling, the expression of TIPE1 has no obvious change, indicating that TIPE1 is one of the upstream target genes of Wnt signalling (Fig. S5).

### Overexpression of β‐catenin counteracted the effect of TIPE1 on EMT

**Figure 7:**
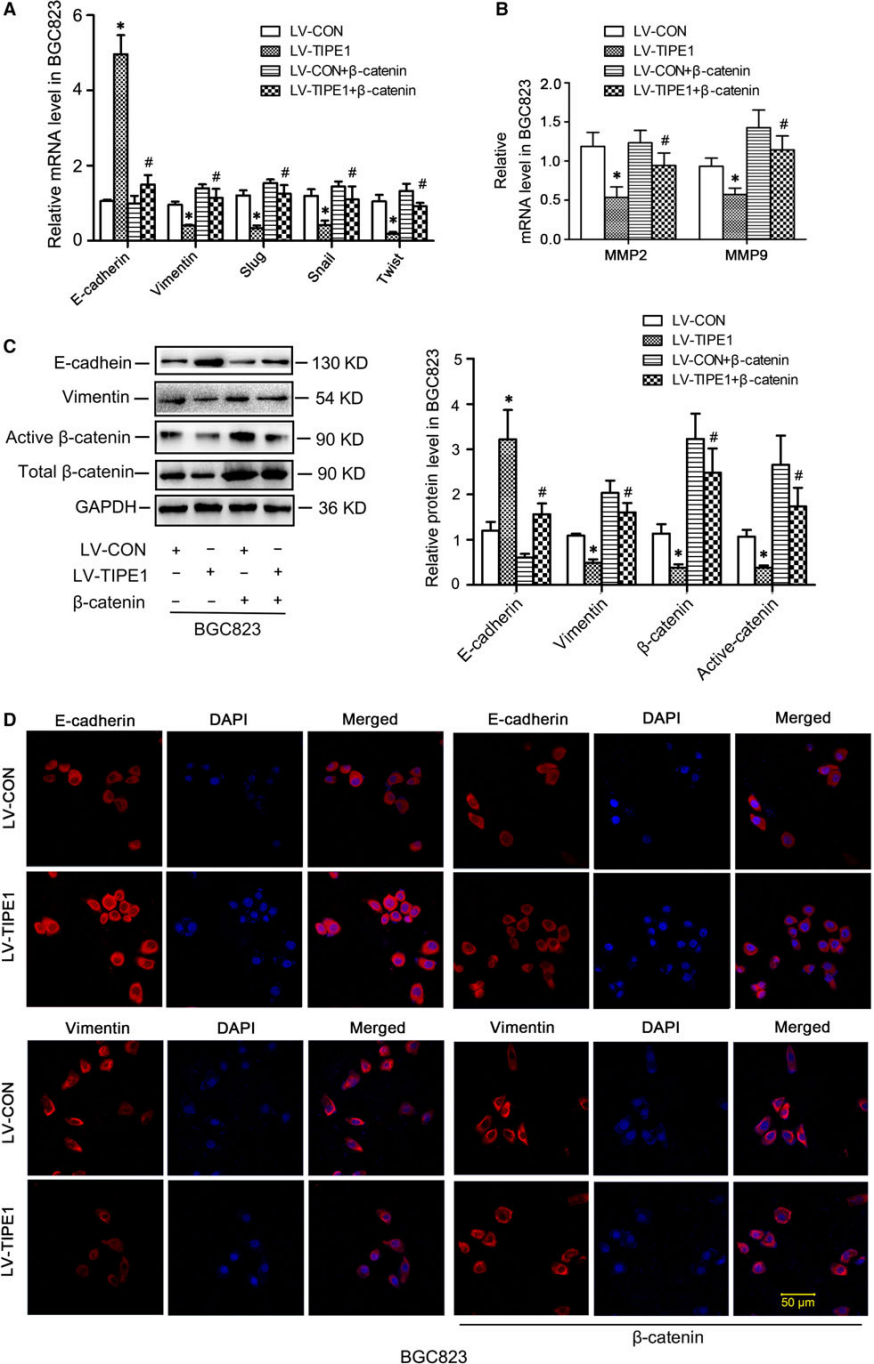
Overexpression of β‐catenin counteracted the effects of TIPE1 on EMT (**A**) Relative mRNA levels of epithelial and mesenchymal markers in BGC823 cells. (**B**) Relative mRNA levels of MMP2 and MMP9 in BGC823 cells. (**C**) Representative Western blot gel documents and summarized data showing the expression levels of E‐cadherin, vimentin, active and total β‐catenin protein in BGC823 cells. (**D**) Immunofluorescence staining results showing the distribution of E‐cadherin and vimentin in BGC823 cells. *P <* *0.05, *versus* LV‐CON groups, ^#^ *P *<* *0.05 *versus* LV‐TIPE1 groups (*n* = 6).

To demonstrate that β‐catenin acts as a key molecular linking TIPE1 and EMT, recombinant vectors pCDNA3.1 harbouring full‐length β‐catenin were transfected into BGC823‐LV‐TIPE1 cells. Western blot analyses confirmed the overexpression of β‐catenin (Fig. 7C) counteracted the effect of TIPE1 on EMT as demonstrated by changed expressions of molecular markers associated with EMT and MMPs in BGC823‐LV‐TIPE1 group (Fig. 7A–D).

### The relative levels of the key molecules of Wnt/β‐catenin signalling in primary gastric cancer specimens

**Figure 8:**
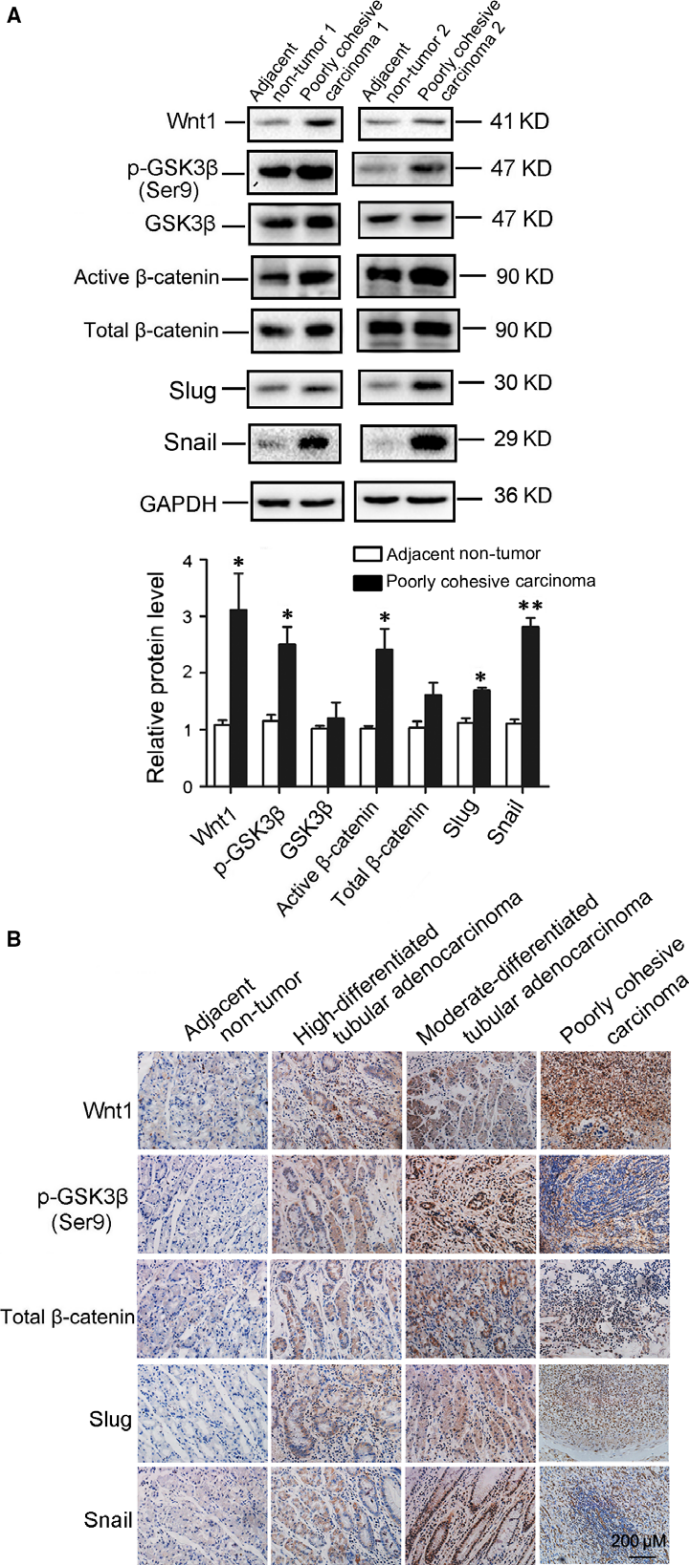
The relative levels of the key molecules of Wnt/β‐catenin signalling were confirmed in primary gastric cancer specimens. (**A**) Representative Western blot gel documents and summarized data showing the expression levels of Wnt1, phosphorylated GSK3β, active β‐catenin, Slug and Snail in poorly cohesive carcinoma tissues. (**B**) Representative immunohistochemical staining showing the expression of Wnt1, phosphorylated GSK3β, active β‐catenin, Slug and Snail in poorly cohesive carcinoma tissues. *P <* *0.05, **P <* *0.01, *versus* adjacent non‐tumour tissues (*n* = 5).

In consistent with the results from *in vitro* study, we found that the levels of Wnt1, phosphorylated GSK3β and active β‐catenin, Slug and Snail were significantly increased in poorly cohesive carcinoma tissues by Western blot (Fig. 8A) and immunohistochemical staining (Fig. 8B) analyses compare with adjacent non‐tumour tissues.

**Figure 9:**
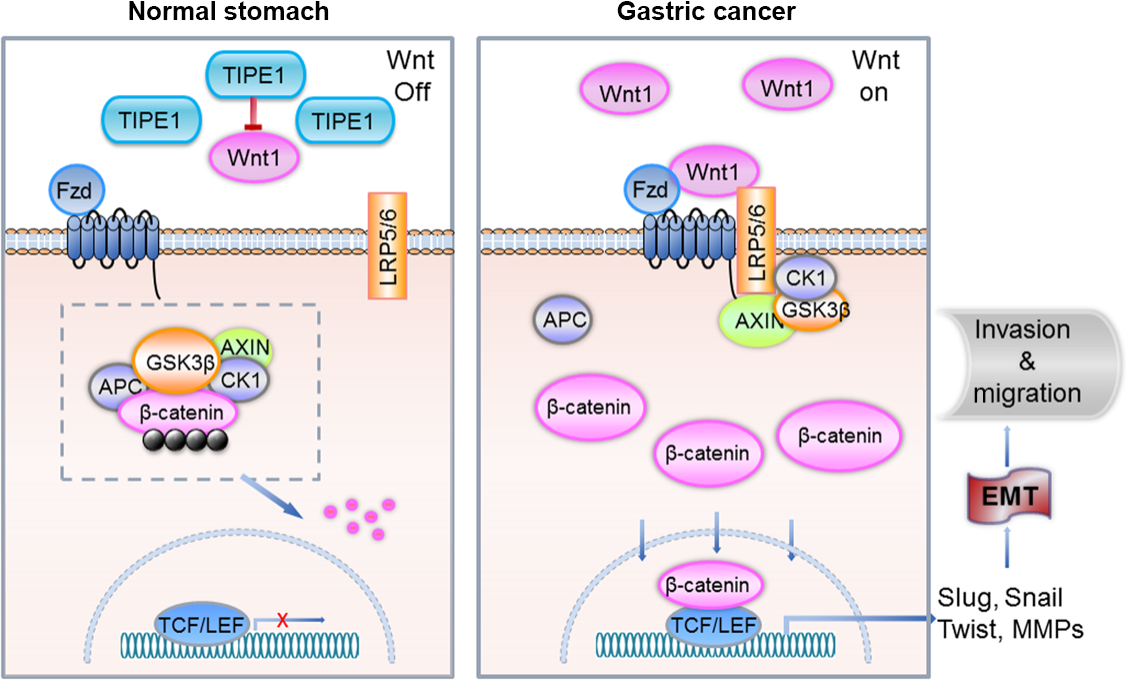
Schematic depicting TIPE1 inhibits EMT, at least in part, *via* negatively regulating Wnt/β‐catenin signalling pathway in gastric cancer. TIPE1, tumour necrosis factor‐alpha‐induced protein‐8 like 1; EMT, epithelial–mesenchymal transition; TCF/LEF, T cell‐specific factors/lymphoid enhancer‐binding factor.

## Discussion

In the present study, we found that the levels of TIPE1 were significantly reduced and were inversely correlated with differentiation status and distant metastasis in patients with primary gastric cancer. Furthermore, we found that TIPE1 negatively regulated cell invasion and metastasis capacities in gastric cancer cells. Mechanistically, we demonstrated that TIPE1 functions as a metastasis suppressor by changing the expression of molecular markers associated with EMT and the activities of MMP2 and MMP9 through, at least in part, inhibiting Wnt/β‐catenin pathway (Fig. 9). This study for the first time demonstrates that TIPE1 plays a functional role in metastasis, suggesting that TIPE1 may serve as a potential therapeutic target for advanced gastric cancer.

Despite the pathogenesis of gastric cancer progression is complex, a growing body of studies highlight the importance of EMT in gastric cancer invasion, metastasis and relapse. Therefore, identifying key molecules involved in EMT in gastric cancer will provide new therapeutic strategy for treating patients with gastric cancer. Emerging evidence has indicated that the TIPE family plays a critical role in tumorigenesis and inflammatory responses. TNFAIP8, the original TIPE family member, is a negative regulator of apoptosis and is considered as an oncogene 7. TIPE2 plays a diverse role in different types of cancers. TIPE2 expression was decreased human hepatic cancer 12 and gastric cancer tissues 13, and reduced TIPE2 expression is associated with metastasis 14. In contrast, TIPE2 expression was increased in the tumour tissues of patients with renal cell carcinoma and positively correlated with TNM staging 15. TIPE3 is the most recently investigated member of the TIPE family. Recent studies have observed that TIPE3 expression was significantly increased in some human cancers such as cervical, colon and lung, and further demonstrated that TIPE3 is an oncogenic transfer protein of lipid second messengers 6, 16. By Agilent Whole Human Genome Oligo Microarray for global human gene expression analysis, we found that among TIPE family, the levels of TIPE1 and TIPE3 were decreased in poorly cohesive gastric carcinoma tissues compared with adjacent non‐tumour tissues. The reduction of TIPE1 in poorly cohesive gastric carcinoma tissues was also confirmed by immunofluorescence staining (Fig. 1B) and Western blot (Fig. 1C). Despite the TIPE family contains four highly homologous mammalian proteins, the unique structure of each member of this family may provide key insights into their functional differences. In this study, we further explored the role of TIPE1 in the development of gastric carcinoma. Although a very recent study revealed that TIPE1 induced apoptosis in hepatocellular carcinoma cells by negatively regulating Rac1 pathway, indicating that the loss of TIPE1 may be a new prognostic indicator for patients with hepatocellular carcinoma 10, we did not observed that TIPE1 significantly induced apoptosis and inhibited cell growth of gastric cancer (Fig. S6). However, we found that TIPE1 could suppress the migration and invasion of gastric cancer through inhibiting EMT. EMT is widely recognized as a key process associating with gastric cancer evolution, during which cancer cells go through phenotypic variations and acquire the capability of migration and invasion. EMT is characterized by the mutative expression of three distinct families of protein consist of cadherins, vimentin and transcription factors including Snail, Twist and Slug 17. An EMT process is distinguished by loss of E‐cadherin, and it creates profound phenotypic alterations through which epithelial cells with apico‐basal polarity convert into front–rear polarity to gain mesenchymal characteristics as well as the capacity of migration, invasion and apoptotic resistance 18. In this study, we found that TIPE1 inhibits EMT as demonstrated by the significant up‐regulation of E‐cadherin expression and downregulation of vimentin and transcription factors in gastric cancer cells. In addition, MMPs especially MMP2 and MMP9 have been demonstrated to promote tumour progression and are implicated in EMT 19, 20. We further found that TIPE1 inhibits the expression of MMP2 and MMP9 and their gelatinolytic activities as well. Taken together, our data strongly support that TIPE1 is a potential suppressor of EMT in gastric cancer.

Mechanistically, we found that TIPE1 negatively regulated Wnt signalling which is an evolutionarily conserved developmental signalling cascade that exhibits a pivotal function in regulating a variety of biologic processes 21, 22, 23. The canonical Wnt pathway is also known as the Wnt/β‐catenin pathway as β‐catenin is a key transducer of the Wnt signal from the cytoplasm to the nucleus. Wnt/β‐catenin pathway has established itself as an EMT regulative signalling due to its maintenance of epithelial integrity as well as tight adherent junctions. Emerging evidence has demonstrated that Wnt/β‐catenin is activated after gastric cancer and plays a critical role in promoting invasion and migration through overexpressing or increasing the functions of several components of the Wnt pathway such as Wnt1, Wnt2, Wnt3, Wnt5a, Fzd‐3, CTNNB1 and LRP6. Owing to the mutuality among EMT and Wnt/β‐catenin pathway in GC pathogenesis, disruption of Wnt/β‐catenin pathway has been shown antimetastatic activity in gastric cancer 24. Although the Wnt/β‐catenin pathway is one of the major signalling pathways involved in EMT, the regulatory mechanisms of it still remain unclear. In this study, one of the most important findings is that we explored the novel biological activities of TIPE1 showing that TIPE1 negatively regulates Wnt/β‐catenin signalling pathways. We found TIPE1 inhibited the expression of Wnt1, a secreted ligand that activates Wnt signalling pathways, which strongly correlates with tumorigenesis and progression 23, 25. Activation of Wnt signalling pathway can promote β‐catenin, a key regulator of the Wnt pathway, nuclear translocation through increasing phosphate GSK3β 26. Indeed our results showed the amount of phosphorylated GSK3β and active β‐catenin (dephosphorylated β‐catenin) was dramatically inhibited by TIPE1. Furthermore, nuclear β‐catenin binds members of the TCF/LEF family transcription factors that activate downstream gene transcription including Slug, Snail and the matrix‐remodelling enzymes 27. Consistently, our data further showed that TIPE1 could also suppress EMT through up‐regulated E‐cadherin binding β‐catenin in the membrane. These findings provide new evidence for a better understanding of the biological activities and regulatory mechanisms of TIPE1 in gastric cancer migration and invasion. It should be noted that the mechanisms by which TIPE1 negatively regulates Wnt1 expression need to be further clarified. Collectively, we found that TIPE1 inhibits gastric cancer invasion and migration, suggesting that TIPE1 may be an innovative therapeutic strategy for treating patients with gastric cancer.

## Conflict of interest

The authors confirm that there are no conflict of interests.

## Supporting information


**Figure S1** Overexpression and gene silencing efficiency of TIPE1 in different gastric cancer cells.
**Figure S2** Representative photographs from different groups of BGC823 cells.
**Figure S3** Immunofluorescence staining results showing the distribution of E‐cadherin and Vimentin in BGC823 cells.
**Figure S4** Gene silencing of TIPE1 on the Wnt/β‐catenin signaling and invasion in AGS cells.
**Figure S5** The expression levels of TIPE1 in AGS with gene silencing of β‐catenin.
**Figure S6** Detection of apoptosis and cell growth of gastric cancer cells.
**Table S1** Primer pairs of target genes used for real time PCR in this study.
**Table S2** Antibodies used in this study.
**Table S3** Constructed sequences used in this study.

## References

[1] Bertuccio P , Chatenoud L , Levi F , *et al* Recent patterns in gastric cancer: a global overview. Int J Cancer. 2009; 125: 666–73. 19382179 10.1002/ijc.24290

[2] Bray F , Jemal A , Grey N , *et al* Global cancer transitions according to the Human Development Index (2008‐2030): a population‐based study. Lancet Oncol. 2012; 13: 790–801. 22658655 10.1016/S1470-2045(12)70211-5

[3] Yang J , Mani SA , Donaher JL , *et al* Twist, a master regulator of morphogenesis, plays an essential role in tumor metastasis. Cell. 2004; 117: 927–39. 15210113 10.1016/j.cell.2004.06.006

[4] Yuan H , Kajiyama H , Ito S , *et al* ALX1 induces snail expression to promote epithelial‐to‐mesenchymal transition and invasion of ovarian cancer cells. Cancer Res. 2013; 73: 1581–90. 23288509 10.1158/0008-5472.CAN-12-2377

[5] Mani SA , Guo W , Liao MJ , *et al* The epithelial‐mesenchymal transition generates cells with properties of stem cells. Cell. 2008; 133: 704–15. 18485877 10.1016/j.cell.2008.03.027 PMC2728032

[6] Fayngerts SA , Wu J , Oxley CL , *et al* TIPE3 is the transfer protein of lipid second messengers that promote cancer. Cancer Cell. 2014; 26: 465–78. 25242044 10.1016/j.ccr.2014.07.025 PMC4198483

[7] Kumar D , Gokhale P , Broustas C , *et al* Expression of SCC‐S2, an antiapoptotic molecule, correlates with enhanced proliferation and tumorigenicity of MDA‐MB 435 cells. Oncogene. 2004; 23: 612–6. 14724590 10.1038/sj.onc.1207123

[8] Sun H , Gong S , Carmody RJ , *et al* TIPE2, a negative regulator of innate and adaptive immunity that maintains immune homeostasis. Cell. 2008; 133: 415–26. 18455983 10.1016/j.cell.2008.03.026 PMC2398615

[9] Zhang Y , Wei X , Liu L , *et al* TIPE2, a novel regulator of immunity, protects against experimental stroke. J Biol Chem. 2012; 287: 32546–55. 22859306 10.1074/jbc.M112.348755 PMC3463367

[10] Zhang Z , Liang X , Gao L , *et al* TIPE1 induces apoptosis by negatively regulating Rac1 activation in hepatocellular carcinoma cells. Oncogene. 2015; 34: 2566–74. 25043299 10.1038/onc.2014.208

[11] Mayes DA , Hu Y , Teng Y , *et al* PAX6 suppresses the invasiveness of glioblastoma cells and the expression of the matrix metalloproteinase‐2 gene. Cancer Res. 2006; 66: 9809–17. 17047041 10.1158/0008-5472.CAN-05-3877

[12] Gus‐Brautbar Y , Johnson D , Zhang L , *et al* The anti‐inflammatory TIPE2 is an inhibitor of the oncogenic Ras. Mol Cell. 2012; 45: 610–8. 22326055 10.1016/j.molcel.2012.01.006 PMC3299909

[13] Zhao Q , Zhao M , Dong T , *et al* Tumor necrosis factor‐alpha‐induced protein‐8 like‐2 (TIPE2) upregulates p27 to decrease gastic cancer cell proliferation. J Cell Biochem. 2015; 116: 1121–9. 25536447 10.1002/jcb.25068

[14] Liu QQ , Zhang FF , Wang F , *et al* TIPE2 inhibits lung cancer growth attributing to promotion of apoptosis by regulating some apoptotic molecules expression. PLoS One. 2015; 10: e0126176. 25946186 10.1371/journal.pone.0126176 PMC4422750

[15] Zhang Z , Qi H , Hou S , *et al* TIPE2 mRNA overexpression correlates with TNM staging in renal cell carcinoma tissues. Oncol Lett. 2013; 6: 571–5. 24137373 10.3892/ol.2013.1388 PMC3789022

[16] Cui J , Hao C , Zhang W , *et al* Identical expression profiling of human and murine TIPE3 protein reveals links to its functions. J Histochem Cytochem. 2015; 63: 206–16. 25479791 10.1369/0022155414564871 PMC4340736

[17] Hao J , Zhang Y , Deng M , *et al* MicroRNA control of epithelial‐mesenchymal transition in cancer stem cells. Int J Cancer. 2014; 135: 1019–27. 24500893 10.1002/ijc.28761

[18] Nilsson GM , Akhtar N , Kannius‐Janson M , *et al* Loss of E‐cadherin expression is not a prerequisite for c‐erbB2‐induced epithelial‐mesenchymal transition. Int J Oncol. 2014; 45: 82–94. 24807161 10.3892/ijo.2014.2424 PMC4079157

[19] Kessenbrock K , Plaks V , Werb Z . Matrix metalloproteinases: regulators of the tumor microenvironment. Cell. 2010; 141: 52–67. 20371345 10.1016/j.cell.2010.03.015 PMC2862057

[20] Yoo YA , Kang MH , Lee HJ , *et al* Sonic hedgehog pathway promotes metastasis and lymphangiogenesis *via* activation of Akt, EMT, and MMP‐9 pathway in gastric cancer. Cancer Res. 2011; 71: 7061–70. 21975935 10.1158/0008-5472.CAN-11-1338

[21] Fu Y , Sun Y , Zhou M , *et al* Therapeutic potential of progranulin in hyperhomocysteinemia‐induced cardiorenal dysfunction. Hypertension. 2017; 69: 259–66. 27872232 10.1161/HYPERTENSIONAHA.116.08154

[22] Liu ZJ , Liu HL , Zhou HC , *et al* TIPE2 Inhibits Hypoxia‐Induced Wnt/beta‐Catenin pathway activation and EMT in Glioma cells. Oncol Res. 2016; 24: 255–61. 27656836 10.3727/096504016X14666990347356 PMC7838627

[23] Mao J , Fan S , Ma W , *et al* Roles of Wnt/beta‐catenin signaling in the gastric cancer stem cells proliferation and salinomycin treatment. Cell Death Dis. 2014; 5: e1039. 24481453 10.1038/cddis.2013.515 PMC4040703

[24] Hanaki H , Yamamoto H , Sakane H , *et al* An anti‐Wnt5a antibody suppresses metastasis of gastric cancer cells *in vivo* by inhibiting receptor‐mediated endocytosis. Mol Cancer Ther. 2012; 11: 298–307. 22101459 10.1158/1535-7163.MCT-11-0682

[25] Zhang H , Xue Y . Wnt pathway is involved in advanced gastric carcinoma. Hepatogastroenterology. 2008; 55: 1126–30. 18705344

[26] Ryu YK , Lee YS , Lee GH , *et al* Regulation of glycogen synthase kinase‐3 by thymosin beta‐4 is associated with gastric cancer cell migration. Int J Cancer. 2012; 131: 2067–77. 22328534 10.1002/ijc.27490

[27] Woo DK , Kim HS , Lee HS , *et al* Altered expression and mutation of beta‐catenin gene in gastric carcinomas and cell lines. Int J Cancer. 2001; 95: 108–13. 11241321 10.1002/1097-0215(20010320)95:2<108::aid-ijc1019>3.0.co;2-#

