# Supplementary material for: TIPE1 suppresses invasion and migration through down‐regulating Wnt/β‐catenin pathway in gastric cancer
Source: J Cell Mol Med. 2017 Oct 10;22(2):1103–17. doi: 10.1111/jcmm.13362 (PMC5783849; doi:10.1111/jcmm.13362)
Supplement: Supplementary file 1 — Figure S1 Overexpression and gene silencing efficiency of TIPE1 in different gastric cancer cells. Figure S2 Representative photographs from different groups of BGC823 cells. Figure S3 Immunofluorescence staining results showing the distribution of E‐cadherin and Vimentin in BGC823 cells. Figure S4 Gene silencing of TIPE1 on the Wnt/β‐catenin signaling and invasion in AGS cells. Figure S5 The expression levels of TIPE1 in AGS with gene silencing of β‐catenin. Figure S6 Detection of apoptosis and cell growth of gastric cancer cells. Table S1 Primer pairs of target genes used for real time PCR in this study. Table S2 Antibodies used in this study. Table S3 Constructed sequences used in this study. [file JCMM-22-1103-s001.doc]

**Electronic Supplementary MaterialS**

**TIPE1 suppresses invasion and migration through down-regulating Wnt/β-catenin pathway in gastric cancer**

Wenwen Liu^1^, Ye Chen^1^, Hua Xie^2^,Yongmin Guo^3^,Dandan Ren^1^, Yupeng Li^4^,Xu Jing^1^, Dongliang Li^3^，Xiao Wang^5^, Miaoqing Zhao^6^, Tianfeng Zhu^1^, Ziying Wang^1^, Xinbing Wei^1^, Fei Gao^7^, Xiaojie Wang^1^, Suxia Liu^8^, Yan Zhang^1#^ and Fan Yi^1#^

^1^Department of Pharmacology, Shandong University School of Medicine, Jinan, China, 250012

^2^Taishan District Center for Disease Control and Prevention, Taian, China, 271000,

^3^Department of Anesthesiology, Qilu Hospital of Shandong University, Jinan, China, 250012

^4^Department of Pediatrics, Peoples Hospital of Rizhao, Rizhao, China, 276800

^5^Department of Pathology, Shandong University School of Medicine, Jinan, China, 250012

^6^Department of Pathology, Shandong Provincial Hospital, Shandong University, Jinan, China, 250021

^7^Key Laboratory of Cardiovascular Remodeling and Function Research, Chinese Ministry of Education and Chinese Ministry of Health, Qilu Hospital, Shandong University, Jinan, China, 250012

^8^Department of Immunology, Shandong University School of Medicine, Jinan, China, 250012

Running title: TIPE1 and EMT in gastric cancer

Words count: 2977

^#^Send Correspondence and Reprint Requests to:

Fan Yi or Yan Zhang

Department of Pharmacology

Shandong University School of Medicine

44#, Wenhua Xi Road,

Jinan, Shandong, 250012, P.R. China

Phone : 86-0531-88382616

Fax : 86-0531-88382616

E-mail: [fanyi@sdu.edu.cn](mailto:fanyi@sdu.edu.cn) or [zhangyan1978@sdu.edu.cn](mailto:zhangyan1978@sdu.edu.cn)

**Materials and Methods**

**Cell transfection:** TIPE1 overexpression lenti-virus and shRNA targeting TIPE1 were obtained from Jikai Gene of Shanghai. The shRNA for TIPE1 or scramble were transfected into cells by Lipofectamine 2000 (Invitrogen) according to the manufacturer’s instructions. TIPE1 overexpression lentivirus was infected into SGC7901 and BGC823 cells.

**RNA extraction and real time RT-PCR:** Total RNA was isolated from tissue or cells using TRIzol reagent (Invitrogen, Life technologies, USA) as described previously . The mRNA levels of target genes were analyzed by real-time RT-PCR using a Bio-Rad iCycler system (Bio-Rad, Hercules, CA) and normalized to β-actin. The specific primers used in this study were shown in Table S1.

**Western blot analysis:** Total cellular lysates preparation and Western blot analysis were performed as described previously . Antibodies used in this study were summarized in Table S2. Rabbit anti-TIPE1 for Western blot was kindly provided by Dr. Suxia Liu (Shandong University,Jinan, China), which has been used in previous studies . To document the loading controls, the membrane was reprobed with a primary antibody against housekeeping protein β-actin or GAPDH.

**Immunohistochemistry:** Tissues from human gastric cancer resected pairs were embedded with paraffin and sliced into 4-μm pieces. The slides were further incubated with the antibodies. The results were detected with diaminobenzidine staining (GTVision™ III Detection System Mo&Rb，Gene Tech, Shanghai PR China) under a microscope (Olympus BX60, Tokyo) and images were captured for analysis as described previously .

**Cell migration and invasion assays:** Cell migration and invasion assays were performed as previously described . Briefly for the migration assays, 8~10×10^5^/ml cells in serum-free media were placed into the upper chamber of an insert (8-μm pore size; Millipore). For the invasion assays, 1×10^6^/ml cells in serum-free medium were placed into the upper chamber of an insert coated with Matrigel (BD). Medium containing 20 % FBS was added to the lower chamber. After incubation for 24 h, the cells remaining on the upper membrane were removed with cotton wool. Cells that had migrated or invaded through the membrane were stained with methanol and 0.1 % crystal violet, imaged, and counted using an inverted microscope (Olympus, Tokyo, Japan).

**References**

1. **Zhang H, Zhu T, Liu W, Qu X, Chen Y, Ren P, Wang Z, Wei X, Zhang Y, Yi F.** TIPE2 acts as a negative regulator linking NOD2 and inflammatory responses in myocardial ischemia/reperfusion injury. *J Mol Med.* 2015; 93: 1033-43.

2. **Yi F, Zhang AY, Janscha JL, Li PL, Zou AP.** Homocysteine activates NADH/NADPH oxidase through ceramide-stimulated Rac GTPase activity in rat mesangial cells. *Kidney Int*. 2004; 66: 1977-87.

3. **Cui J, Zhang G, Hao C, Wang Y, Lou Y, Zhang W, Wang J, Liu S.** The expression of TIPE1 in murine tissues and human cell lines. *Mol Immunol.* 2011; 48: 1548-55.

4. **Zhang Y, Wei X, Liu L, Liu S, Wang Z, Zhang B, Fan B, Yang F, Huang S, Jiang F, Chen YH, Yi F.** TIPE2, a novel regulator of immunity, protects against experimental stroke. *J Biol Chem.* 2012; 287: 32546-55.

5. **Gohrig A, Detjen KM, Hilfenhaus G, Korner JL, Welzel M, Arsenic R, Schmuck R, Bahra M, Wu JY, Wiedenmann B, Fischer C.** Axon guidance factor SLIT2 inhibits neural invasion and metastasis in pancreatic cancer. *Cancer res.* 2014; 74: 1529-40.

**Table S1. Primer pairs of target genes used for real time PCR in this study**

| **Genes** | **Accession No.** | **Forward** | **Reverse** |
| --- | --- | --- | --- |
| Homo β-actin | [XM_006715764.1](http://www.ncbi.nlm.nih.gov/entrez/viewer.fcgi?db=nucleotide&id=578813593) | CACTGTGTTGGCGTACAGGT | TCATCACCATTGGCAATGAG |
| Homo TIPE1 | NM_152362 | TGCTCAAGAACCTGGTCAAGG | AGGAAGTCGCAGTCGGCTA |
| Homo E-cadheirn | [NM_001317186.1](http://www.ncbi.nlm.nih.gov/entrez/viewer.fcgi?db=nucleotide&id=953768351) | TTCCTCCCAATACATCTCCC | TTGATTTTGTAGTCACCCACC |
| Homo Vimentin | NM_003380.3 | CTCTTCCAAACTTTTCCTCCC | AGTTTCGTTGATAACCTGTCC |
| Homoβ-catenin | NM_001098209 | AAAGCGGCTGTTAGTCACTGG | CGAGTCATTGCATACTGTCCAT |
| Homo Slug | [NM_003068.4](http://www.ncbi.nlm.nih.gov/entrez/viewer.fcgi?db=nucleotide&id=324072669) | CGCCTCCAAAAAGCCAAAC | CGGTAGTCCACACAGTGATG |
| Homo Snail | [NM_005985.3](http://www.ncbi.nlm.nih.gov/entrez/viewer.fcgi?db=nucleotide&id=301336132) | GGAAGCCTAACTACAGCGAGCT | TCCCAGATGAGCATTGGCA |
| Homo Twist | [NM_000474.3](http://www.ncbi.nlm.nih.gov/entrez/viewer.fcgi?db=nucleotide&id=68160957) | GTCCGCAGTCTTACGAGGAG | GCTTGAGGGTCTGAATCTTGCT |
| Homo MMP2 | [NM_001302510.1](http://www.ncbi.nlm.nih.gov/entrez/viewer.fcgi?db=nucleotide&id=700274114) | AGAGACAGTGGATGATGCCTTT | ATCGTCATCAAAATGGGAGTCT |
| Homo MMP9 | [NM_004994.2](http://www.ncbi.nlm.nih.gov/entrez/viewer.fcgi?db=nucleotide&id=74272286) | TGTACCGCTATGGTTACACTCG | GGCAGGGACAGTTGCTTCT |
| Homo Wnt1 | NM_005430.3 | TCCCCTTTGTCCTGCGTTTT | CCCCCAACCTCATTTCCACA |
| Homo Wnt2 | [NM_003391.2](http://www.ncbi.nlm.nih.gov/entrez/viewer.fcgi?db=nucleotide&id=195230749) | CACACGCTGCACCTAAAGC | AATTACCCCTAAGGGTGGTAGC |
| Homo Wnt3 | [NM_030753.4](http://www.ncbi.nlm.nih.gov/entrez/viewer.fcgi?db=nucleotide&id=459683869) | CAGCAGTACACATCTCTGGGCTCA | CTGTCATCTATGGTGGTGCAGTTC |
| Homo Wnt3a | [NM_033131.3](https://www.ncbi.nlm.nih.gov/entrez/viewer.fcgi?db=nucleotide&id=325053704) | GCCTCGGAGATGGTGGTG | GTTGGGCTCGCAGAAGTTG |
| Homo Wnt5a | [NM_003392.4](http://www.ncbi.nlm.nih.gov/entrez/viewer.fcgi?db=nucleotide&id=371506361) | ACTGGCAGGACTTTCTCAAGGACA | GCCTATCTGCATCACCCTGCCAAA |
| Homo Wnt7a | [NM_004625.3](http://www.ncbi.nlm.nih.gov/entrez/viewer.fcgi?db=nucleotide&id=34328912) | AATGCCCGGACTCTCATGAACTT | ACGGCCTCGTTGTACTTGTCCTT |

**Table S2. Antibodies used in this study**

| **Primary antibodies** | **Host** | **Dilution and supplier** | | **Application** |
| --- | --- | --- | --- | --- |
| TIPE1 | Rabbit | 1:100 ; ProteinTech Group, Chicago, IL | | IHC |
| Flag | Mouse | 1:1000;abcam | | WB |
| MMP2 | Rabbit | 1:1000; ProteinTech Group, Chicago, IL | | WB |
| MMP9 | Rabbit | 1:1000; ProteinTech Group, Chicago, IL | | WB |
| Slug | Rabbit | 1:1000; Cell Signaling, Danvers, MA | | WB |
| Snail | Rabbit | 1:1000; Cell Signaling, Danvers, MA | | WB |
| β-catenin | Rabbit | 1:1000; ProteinTech Group, Chicago, IL | | WB,IF |
| Active-β-catenin | Rabbit | 1:1000;CST | | WB |
| E-cadherin | Rabbit | 1:1000; ProteinTech Group, Chicago, IL | | WB,IF |
| Vimentin | Rabbit | 1:1000; ProteinTech Group, Chicago, IL | | WB,IF |
| Wnt1 | Rabbit | 1:1000;abcam | | WB,IHC |
| Wnt2 | Rabbit | 1:1000; ProteinTech Group, Chicago, IL | | WB,IHC |
| Wnt3 | Rabbit | 1:1000; ProteinTech Group, Chicago, IL | | WB |
| Wnt3a | Rabbit | 1:1000；ABclonal | | WB |
| Wnt5a | Rabbit | 1:1000; ProteinTech Group, Chicago, IL | | WB,IHC |
| Wnt7a | Rabbit | 1:1000; ProteinTech Group, Chicago, IL | | WB |
| GSK3β | Rabbit | 1:1000; ProteinTech Group, Chicago, IL | | WB |
| p-GSK3β | Rabbit | 1:5000; abcam | WB | |
| GAPDH | Rabbit | 1:1000; ProteinTech Group, Chicago, IL | | WB |
| β-actin | Rabbit | 1:1000; ProteinTech Group, Chicago, IL | | WB |

**Table S3. Constructed sequences used in this study**

| **Name** | **Sense** | **Antisense** |
| --- | --- | --- |
| siRNA-catenin-1 | ACGACUAGUUCAGUUGCUUDTDT | AAGCAACUGAACUAGUCGUDTDT |
| siRNA-catenin-2 | CCUGGUGAAAAUGCUUGGUDTDT | ACCAAGCAUUUUCACCAGGDTDT |
| siRNA-catenin-3 | GUGCUAUCUGUCUGCUCUADTDT | UAGAGCAGACAGAUAGCACDTDT |
| shRNA-TIPE1-1 | CCCAGAAGAUGCUCAAGAATT | UUCUUGAGCAUCUUCUGGGTT |
| shRNA-TIPE1-2 | GACACCUUCAGCACCAAGATT | UCUUGGUGCUGAAGGUGUCTT |
| shRNA-TIPE1-3 | GAGUAAGAUGGCGUCCAAGTT | CUUGGACGCCAUCUUACUCTT |

**
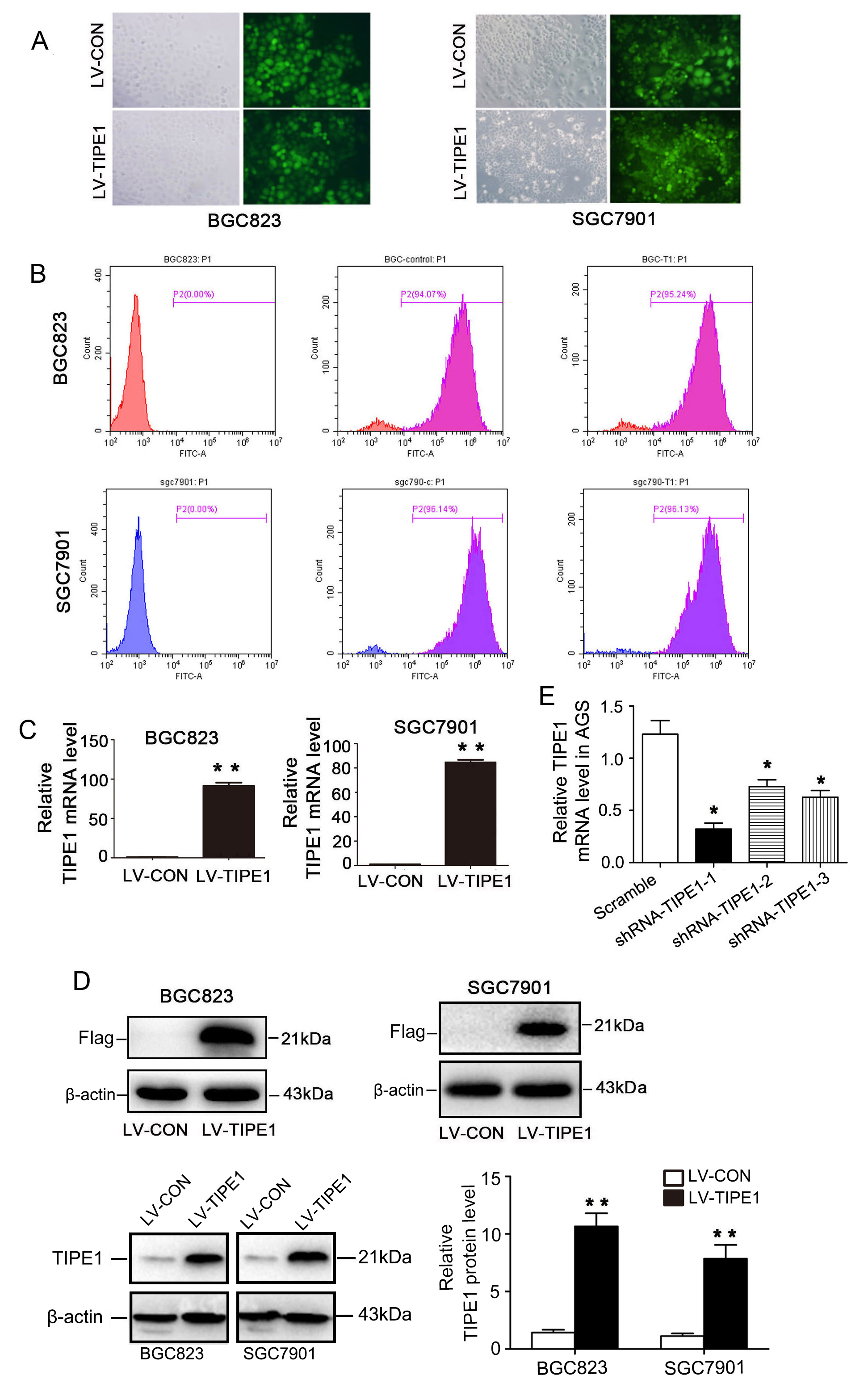
**

**Figure S1. Overexpression and gene silencing efficiency of TIPE1 in different gastric cancer cells:** (A) Representative cell photographs in gastric cancer cells (BGC823 and SGC7901) with TIPE1 lentivirus (LV-TIPE1) transfection. (B) Representative flow cytometry results in gastric cancer cells (BGC823 and SGC7901) with TIPE1 lentivirus (LV-TIPE1) transfection. (C) Relative mRNA levels of TIPE1 in in gastric cancer cells (BGC823 and SGC7901) with TIPE1 lentivirus (LV-TIPE1) transfection. (D) Representative Western blot gel documents showing the expression levels of TIPE1 in gastric cancer cells (BGC823 and SGC7901) with TIPE1 lentivirus (LV-TIPE1) transfection. (E) Relative mRNA levels of TIPE1 in BGC823 cells transfecting three different sets of shRNA-TIPE1. The results showed shRNA-TIPE1-1 had more efficiency on gene silencing of TIPE1, which was used for further studies. * *P* < 0.05, ** *P* < 0.01 vs. LV-CON or scramble groups (n=5)


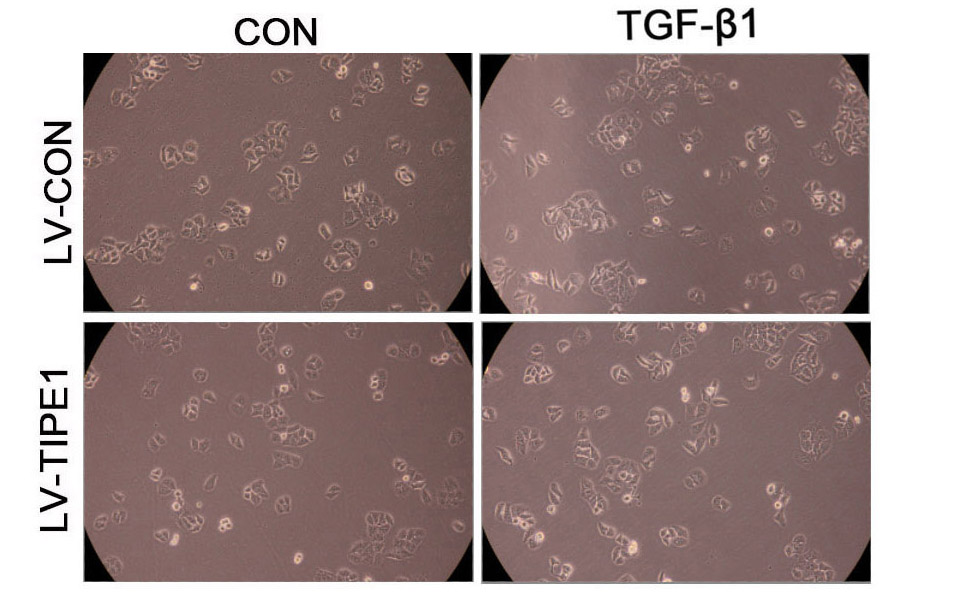


**Figure S2.** Representative photographs from different groups of BGC823 cells


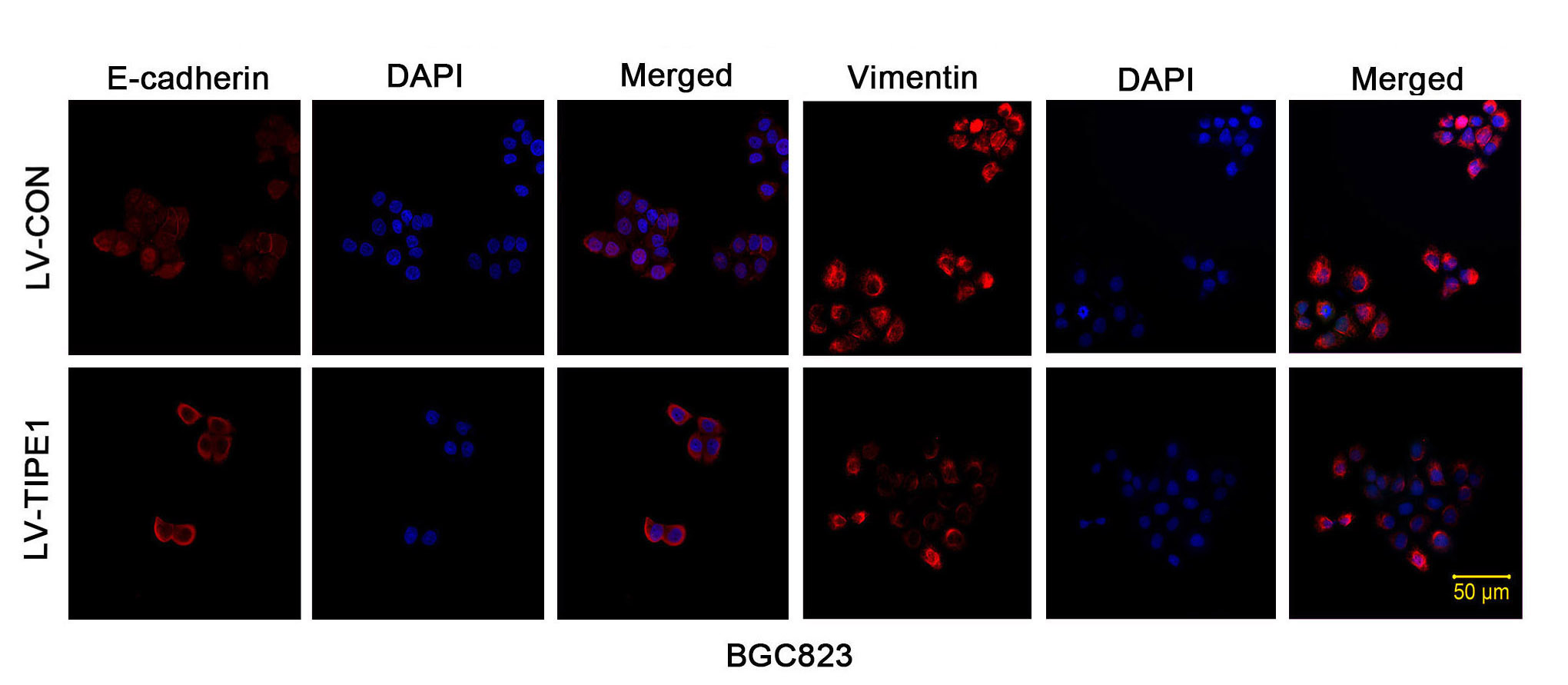


**Figure S3.** Immunofluorescence staining results showing the distribution of E-cadherin and Vimentin in BGC823 cells.


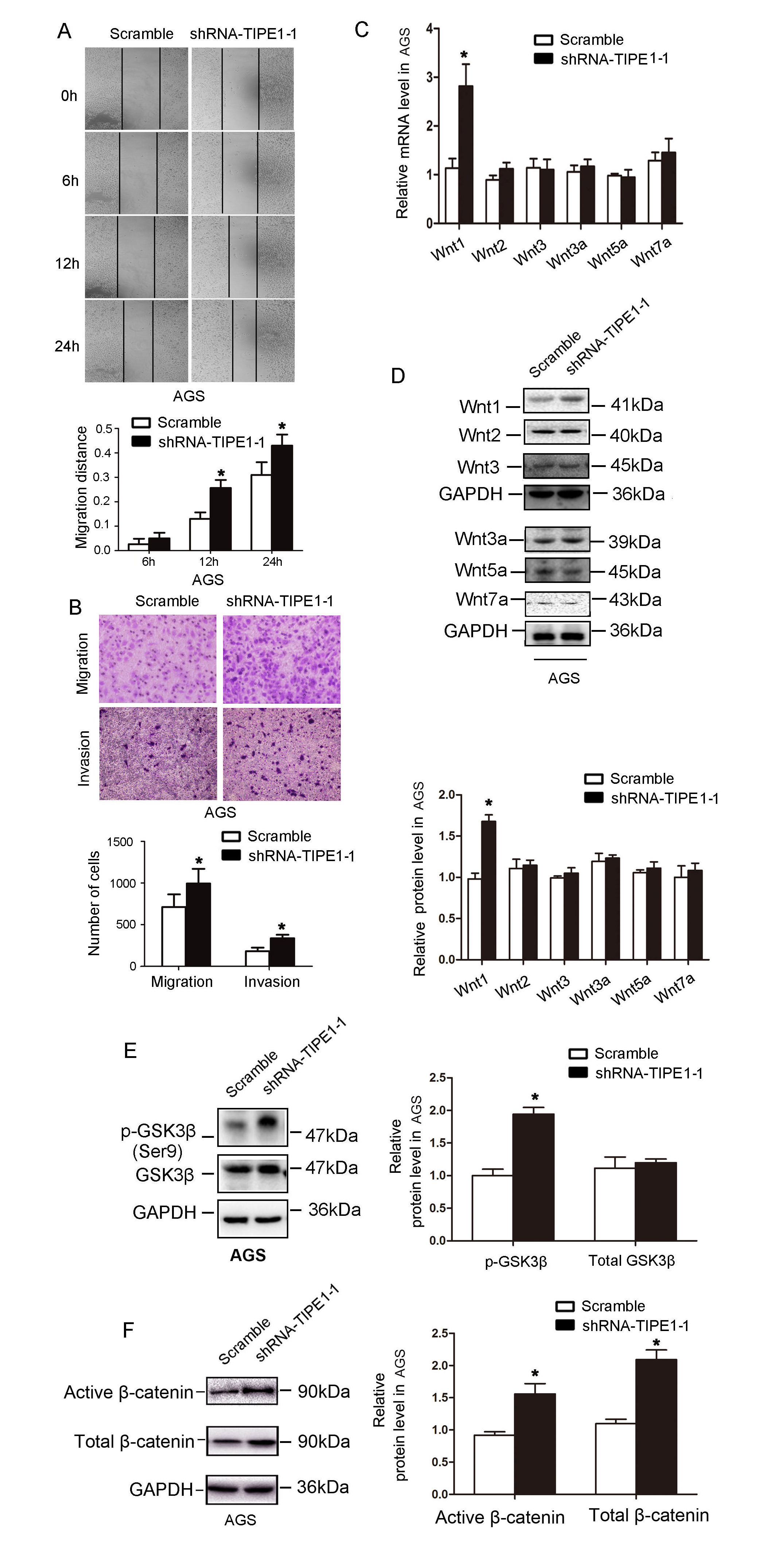


**Figure S4. Gene silencing of TIPE1 on the Wnt/β-catenin signaling and invasion in AGS cells:** (A) Representative and quantification of wound healing assay in AGS cells with gene silencing of TIPE1. (B) Representative migration and invasion assay in AGS cells with gene silencing of TIPE1 and summarized data showing the number of cells passing through the Matrigel filter. (C) Relative mRNA levels of TIPE1, Wnt1, Wnt2, Wnt3, Wnt3a, Wnt5a and Wnt7a in AGS cells with gene silencing of TIPE1. (D) Representative Western blot gel documents and summarized data showing the expression levels of Wnt1, Wnt2, Wnt3, Wnt3a, Wnt5a and Wnt7a in AGS cells with gene silencing of TIPE1. (E) Representative Western blot gel documents and summarized data showing the expression levels of total and phosphorylated GSK3β protein in AGS cells with gene silencing of TIPE1. (F) Representative Western blot gel documents and summarized data showing the expression levels of active and total β-catenin protein in AGS cells with gene silencing of TIPE1.* *P* < 0.05, vs. scramble groups (n=5)


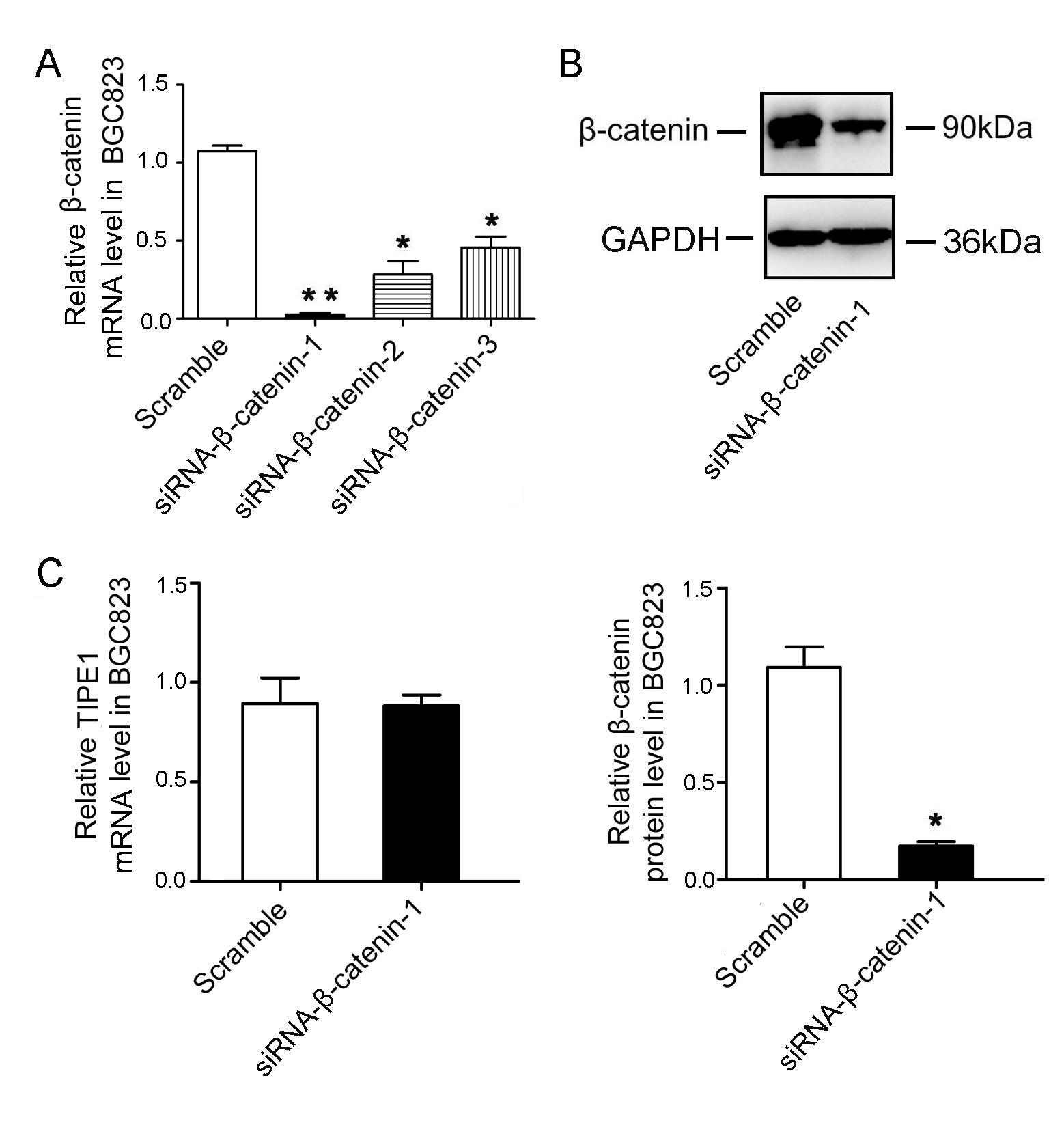


**Figure S5. The expression levels of TIPE1 in AGS with gene silencing of β-catenin:** (A) Relative mRNA levels of β-catenin in BGC823 cells transfecting three different sets of shRNA-β-catenin. The results showed shRNA-β-catenin -1 had more efficiency on gene silencing of β-catenin, which was used for further studies. (B) Representative Western blot gel documents and summarized data showing the expression levels of total β-catenin protein in BGC823 cells transfecting siRNA-β-catenin-1. (C) Relative mRNA levels of TIPE1 in BGC823 cells after transfecting siRNA-β-catenin-1. * *P* < 0.05, * **P* < 0.01 vs. scramble groups (n=5)


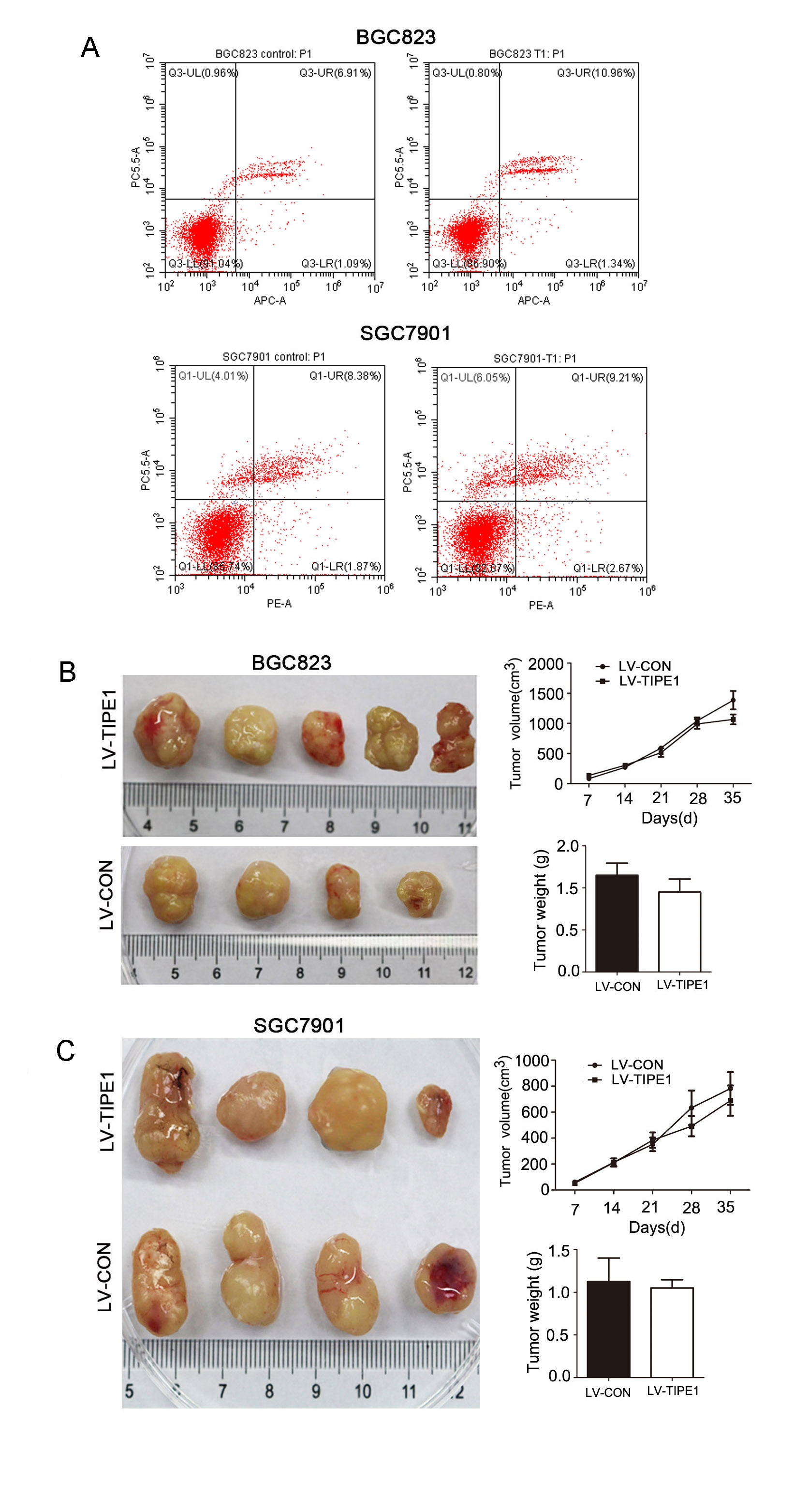


**Figure S6. Detection of apoptosis and cell growth of gastric cancer cells:** Representative apoptosis results in different groups of gastric cancer cells (B) Representative photographs, tumor volume and tumor weight of different groups of BGC823 cells. (C) Representative photographs, tumor volume and tumor weight of different groups of SGC7901 cells. (n=10)
